# An investigation of a novel milk allergy-friendly food supplement program

**DOI:** 10.3389/falgy.2024.1301834

**Published:** 2024-06-18

**Authors:** Michael A. Golding, Manvir Bhamra, Zoe Harbottle, Moshe Ben-Shoshan, Jennifer D. Gerdts, Leslie E. Roos, Elissa M. Abrams, Sara J. Penner, Jo-Anne St-Vincent, Jennifer L. P. Protudjer

**Affiliations:** ^1^ The Children’s Hospital Research Institute of Manitoba, Winnipeg, MB, Canada; ^2^ Department of Pediatrics and Child Health, University of Manitoba, Winnipeg, MB, Canada; ^3^ Department of Food and Human Nutritional Sciences, University of Manitoba, Winnipeg, MB, Canada; ^4^ Department of Allergy and Immunology, McGill University, Montreal, QC, Canada; ^5^ Food Allergy Canada, Toronto, ON, Canada; ^6^ Department of Psychology, University of Manitoba, Winnipeg, MB, Canada; ^7^ Department of Pediatrics, Faculty of Medicine, University of British Columbia, Vancouver, BC, Canada; ^8^ Department of Business and Administration, University of Winnipeg, Winnipeg, MB, Canada; ^9^ Children’s Allergy and Asthma Education Centre, Winnipeg, MB, Canada; ^10^ George and Fay Yee Centre for Healthcare Innovation, Winnipeg, MB, Canada; ^11^ Institute of Environmental Medicine, Karolinska Institutet, Stockholm, Sweden

**Keywords:** food hypersensitivity, cost of illness/food hypersensitivity, dairy allergy, intervention, quality of life, food security

## Abstract

**Introduction:**

Compared to households not managing food allergy, households managing food allergy are faced with greater direct and indirect costs. To address these cost burdens, we developed and piloted a milk allergy-friendly food supplement program for lower- and middle-income households managing a dairy allergy in a child age <6 years. Herein, we aimed to evaluate to the impact of this program on food costs, food security, and caregiver mental health using a longitudinal design.

**Methods:**

Participants living in or near the city of Winnipeg, in Manitoba, Canada were recruited from January to February 2022 via social media, word-of-mouth, and a database maintained by the principal investigator. Consenting participants took part in a 6-month allergen-friendly food supplement program that provided them with biweekly deliveries of allergen-friendly foods free of charge. To evaluate the impact of the program on food costs, food security, and well-being, participants completed a series of questionnaires at baseline, mid-point, and at the end of the program. Changes in these variables were assessed via a series of Friedman tests.

**Results:**

The final sample was comprised of 8 households. Relative to baseline, participants reported higher total direct food costs at midpoint (+5.6%) and endpoint (+13.5%), but these changes did not reach statistical significance. In contrast, total indirect food costs decreased over the course of the study relative to baseline (midpoint = −28.2%; endpoint = −18.5%), but the changes were not found to be statistically significant. Participants did, however, report a statistically significant decrease in costs related to lost time from work or school as a result of their child's food allergy at endpoint relative to baseline (−100%). Few changes in food security, caregiver well-being, or child food allergy quality of life were noted.

**Discussion:**

The provision of allergen-friendly foods helped keep grocery costs below the pace of inflation. Participants also reported reduced costs associated with missed time from work or school as a result of their child's food allergy. Despite these encouraging findings, a relatively high proportion of the current sample reported experiencing food insecurity throughout the study period, suggesting that additional financial support for families is needed.

## 1 Introduction

Food allergy is a common pediatric health condition that has been found to affect as many as 10% of children in some Western countries (1). While immunotherapies provide a promising means to increase the ability of some children to tolerate exposure to their food allergen, the primary treatment for the majority of individuals with food allergy remains total avoidance of the offending food. Naturally, complete avoidance of a particular food or ingredient can impose a considerable burden on individuals with food allergy and their caregivers (2, 3). Not surprisingly, some of this burden can be attributed to worries surrounding accidental exposures and social impediments caused by dietary restrictions (2, 3). However, households managing food allergy also report being burdened financially. In fact, a 2021 review comparing families with and without a child with food allergy, found families with a child with food allergy had a median out-of-pocket cost differential of +$1,707.46 US dollars (December 2020) per year (IQR = $1,026.72–$2,115.79) (2). In addition to out-of-pocket expenses, households with a member with food allergy often report higher indirect costs (e.g., lost time and productivity), as a result of increased food preparation, shopping time and greater levels of healthcare utilization (4). Moreover, the need for emergency care and routine follow-up appointments has also been found to impact the careers of some parents of children with food allergy through increased absenteeism and reduced productivity (5–7).

A small, but emerging body of research suggests that these additional costs may leave some food-allergic households more vulnerable to experiencing food insecurity. Research by Dilley and colleagues found elevated rates of food insecurity were limited to children with *both* egg and milk allergy; however, other studies have provided evidence that food insecurity is roughly 30% more prevalent among food-allergic families in general (8–10). More recent research suggests that this discrepancy between households with food allergy and those without was even greater during the early months of the COVID-19 pandemic as individuals with food allergy were 60% more likely to report new or worsening food insecurity following the outbreak of the pandemic relative to those without dietary restrictions (8).

While individuals managing food allergy incur additional costs as a result of their condition, little financial support is currently available in Canada. Interestingly, Canadians with celiac disease are eligible to claim the difference in cost between gluten-free foods and foods containing gluten on their federal income tax; however, this same benefit is not extended to individuals with food allergy (11). In light of these gap, we developed and piloted a dairy allergy-friendly food supplement program that provided lower and middle-income families managing a pediatric dairy allergy in Winnipeg, Manitoba with biweekly donations of allergen-friendly foods. Herein, we aimed to evaluate the impact of this program on food costs, food security, and caregiver mental health using a longitudinal design.

## 2 Methods

### 2.1 Recruitment

Participants were recruited from January to February 2022 through a database maintained by the principal investigator, word-of-mouth and Winnipeg-based social media groups focused on individuals managing food allergy. As a pilot project, the current study was limited to parents with a child under the age of 6 years old with a physician-diagnosed dairy allergy. Dairy allergy was selected for the current study as it is prevalent among children and has been described as particularly burdensome given the ubiquity of milk (1, 12). Eligible food allergies included both Immunoglobulin E (IgE) and non-IgE immune-mediated allergies. Children with non-immune-mediated intolerances were not eligible for the current study. Participants were asked to confirm their eligibility by providing a proof of allergy letter from the child's pediatrician or allergist. Participants were, however, reimbursed for any costs they incurred in obtaining the letter. The current study was also limited to households living in or near the city of Winnipeg, Manitoba with an annual, after-tax, household income of $70,000 Canadian dollars (i.e., CAD) or less in the year prior to recruitment.

### 2.2 Intervention

Consenting families participated in a six-month, allergen-friendly food supplement program that provided them with packages of food products and coupons every two weeks for a period of six-months. Each package was valued at approximately $50.00 CAD and contained items donated by Daiya, a producer of plant-based, allergy-friendly foods. All items were free of dairy, wheat, soy, egg, peanut, tree nut, fish, and shellfish. The content of the packages were largely similar across participants, although some substitutions were made based on the household's taste preferences and other food allergies. Throughout the study, the packages were delivered to the participant's home or other mutually agreed upon location by a pair of research assistants.

### 2.3 Data collection

In order to better understand the impact of the allergy-friendly food supplement program, participants were asked to complete a series of questionnaires at three time points throughout the study. The first set of questionnaires was completed approximately two weeks before the delivery of the first food package and was used to establish a baseline of participants’ pre-study functioning and food costs. Participants completed the same set of questionnaires at the mid-point of the study and again two weeks prior to the delivery of the final food package to track their changes in food costs, food security, and mental health throughout the study. All questionnaires were completed online using the REDCap survey software.

### 2.4 Measures

Food-related costs were measured using an adapted version of the Food Allergy Economic Questionnaire (FA-EcoQ), a validated self-report questionnaire designed to measure the direct and indirect costs associated with food allergy (13). Direct costs capture the medical and non-medical expenses associated with the maintenance of one's health that are paid out-of-pocket (14). In the current study, direct medication and food costs (i.e., groceries and prepared meals) were assessed. Indirect costs, on the other hand, include the costs associated with lost time or productivity (14). Herein, indirect costs included those incurred through grocery shopping, meal preparation and lost time from work or school due to child's food allergy. Indirect costs were calculated by multiplying the number of hours lost by the after-tax hourly wage reported by the family member incurring the cost. If an individual was unemployed, their time was valued at the provincial after-tax minimum wage at the time of data analysis ($11.95 CAD). To limit participant burden, several items that were irrelevant to the aims of study were omitted from the FA-EcoQ. A number of items were also modified to better reflect the Canadian vernacular.

In addition to the FA-EcoQ, participants were asked to complete the Household Food Security Survey Module (HFSSM), an 18-item self-report measure of food access and availability, derived from the larger Canadian Community Health Survey (15, 16). Ten of the 18 items are used to measure adult food security, while the remainder center on child food security. In the current study, participants completed both the adult and child scales. In completing the measure, participants are typically asked to rate the degree to which have experienced problems of food access and availability over the previous year. In the current study, however, participants were asked to rate their ability to access food over the previous three months. Based on Health Canada's classification scheme, individuals with less than two affirmative responses on either the child or adult food insecurity scale are considered food secure (17). Moderate food insecurity is indicated by 2–5 affirmative responses on the adult scale and 2–4 affirmative responses on the child scale. Severe food insecurity on the other hand is indicated by 6 or more affirmative responses on the adult scale and 5 or more on the child scale.

Participants also completed several questionnaires related to their mental health, including the Perceived Stress Scale (18), the Generalized Anxiety Disorder 7-item scale (19), and the Center for Epidemiological Studies Depression Scale Revised (20). Lastly, caregivers were asked to assess the target child's food allergy health-related quality of life using the Food Allergy Quality of Life Questionnaire Parent Form (FAQLQ-PF) (21).

Following the completion of the study, participants also completed a semi-structured interview to better understand their thoughts on the program; however, findings from these interviews are presented in separate paper (22).

### 2.5 Data analysis

Data from the current study was described using medians, means, standard deviations, and frequencies. Inferential analyses were also used to determine whether food costs, food security, food allergy quality of life (FA-QoL), and psychosocial health varied across each of the three time points. Because the small sample size precluded an accurate assessment of normality, changes in the outcome variables were assessed using a series of Friedman tests, a non-parametric alternative to the one-way repeated measures ANOVA. As an omnibus test, it requires post-hoc testing to identify which particular groups or treatments differ in the event of a significant result. In the current study, Conover-Iman tests, corrected for multiple comparisons using Bonferroni's method, were used to investigate significant findings from the Friedman test. All analyses were conducted using Stata 17 (College Station, TX), with the exception of the Conover-Iman tests, which were run in R Commander (version 2.8-0). Statistical significance was set at *α* = 0.05 for each of the inferential analyses.

## 3 Results

### 3.1 Demographics

A total of 11 households began the study, but 3 were lost to follow-up before the second time-point and were excluded from the final analyses (*N* = 8). A series of Mann–Whitney *U* and *χ*^2^ tests revealed households who completed the study did not significantly differ on any of the demographic or outcome variables from those who were lost to follow-up.

All of the adults included in the final sample reported being the mother to the child in the intervention. Participants ranged in age from 26 to 39 years and were approximately 30 years old on average (*M* = 29.88, SD = 1.41). The majority (62.5%) of respondents reported having a spouse at the time of the study and half had more than one child. The average after-tax monthly household income was found to be $3,203.14 (CAD; SD = 1,774.50) or $38,437.68 (SD = 21,294) annually. Children participating in the study were ethnically diverse (multiracial: 37.5%; White: 37.5%; other: 25%) and ranged in age from <1–5 years (*M *= 2.06 years; SD = 1.32). Most children had additional food allergies apart from dairy (87.5%). Egg was the most common additional allergy (50%), followed by peanut (37.5%) and soy (37.5%). Most families had only one member with food allergy; however, a sizeable minority did report having an second household member with food allergy (37.5%; Please see Table 1 for complete summary of participant demographics).

**Table 1:** Demographic and clinical characteristics.

	Mean (SD)	%	*n*
Respondent age	29.9 years (4.0)	–	–
Respondent gender			
Female		100.0%	8
Male		0.0%	0
Respondent education			
High school diploma or less		37.5%	3
Post-secondary degree/diploma		62.5%	5
Married or cohabitating			
Yes		62.5%	5
No		37.5%	3
Respondent employment status			
Employed		37.5%	3
Unemployed, not seeking work		37.5%	3
Unemployed, seeking work; Other		25.0%	2
Spouse employment status			
Employed		100.0%	5
Annual after-tax household income	$38,437.68 ($21,294.50)		
Number of adults in household	2.00 (0.95)		
Number of children in household	2.25 (1.58)		
Target child's age	2.1 years (1.3)	–	–
Target child's sex			
Female		37.5%	3
Male		62.5%	5
Target child food allergies^a^			
Dairy		100.0%	8
Egg		50.0%	4
Peanut		37.5%	3
Soy		37.5%	3
Fish		12.5%	1
Tree nut		12.5%	1
Sesame		12.5%	1
Other		25.0%	2
Target child number of food allergies	2.88 (1.25)		
1		12.5%	1
2		25.0%	2
3+		66.5%	5

^a^ Not mutually exclusive.

### 3.2 Substantive analyses

#### 3.2.1 Direct food costs

**Figure 1:**
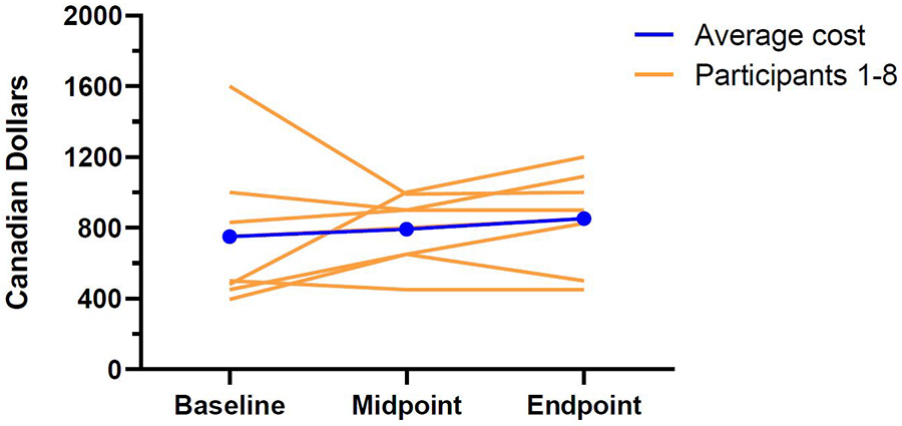
Individual and average direct food costs at baseline, midpoint, and endpoint.

Across the study period, total direct food costs increased by 13.5%, on average (See Table 2 for a summary of the substantive findings). At baseline, participants reported spending an average of $750.75 CAD per month (SD = 404.08) on groceries and prepared meals (See Figure 1 for an illustration of the direct food costs at baseline, midpoint, and endpoint for each participant). At midpoint, average monthly food costs were found to be $792.50 (SD = 193.88) and by the end of the study these costs increased to $851.88 (SD = 264.09). Much of the increased spending on food appeared to stem from restaurant and takeaway meals as spending on prepared meals nearly doubled from baseline (*M *= $63.25, SD = 70.08) to midpoint (*M *= $118.75, SD = 138.71) and remained elevated at the end of the study (*M *= $139.38, SD = 162.71). Despite these apparent differences in direct food costs, none of the changes reached statistical significance. Similarly, changes in monthly medication costs failed to reach statistical significance despite decreasing modestly from baseline (*M *= 5.03, SD = 8.20) to endpoint (*M *= 2.67, SD = 12.51).

**Table 2:** Direct and indirect costs across the study's duration.

	Baseline		Midpoint		Endpoint		Difference	
	Median (IQR)	Mean (SD)	Median (IQR)	Mean (SD)	Median (IQR)	Mean (SD)	Friedman *χ*^2^	*p*
Direct costs^a^								
Total monthly food costs	$625.00 (465.00, 915.00)	$750.75 (404.08)	$850.00 (650.00, 954.00)	$792.50 (193.89)	$875.00 (662.50, 1,045.00)	$851.88 (264.09)	3.47	0.18
Grocery costs	$625.00 (375.00, 800.00)	$687.50 (421.52)	$650.00 (525.00, 825.00)	$673.75 (201.28)	$725.00 (475.00, 950.00)	$712.50 (251.78)	0.90	0.64
Prepared meals	$55.50 (0.00, 97.50)	$63.25 (70.08)	$75.00 (25.00, 175.00)	$118.75 (138.71)	$100.00 (0.00, 425.00)	$139.38 (162.71)	1.00	0.61
Monthly medication costs	$0.00 (0.00, 22.91)	$15.08 (24.61)	$3.34 (0.00, 29.40)	$14.44 (19.35)	$0.00 (0.00, 21.35)	$8.00 (12.51)	4.93	0.08
Indirect costs^a^								
Total monthly food costs	$505.50 (370.45, 717.00)	$574.42 (304.48)	$400.32 (167.30, 669.20)	$412.47 (261.83)	$435.98 (161.72, 701.09)	$468.10 (371.59)	0.92	0.63
Shopping costs	$89.62 (57.72, 123.44)	$99.06 (58.15)	$59.75 (41.82, 107.55)	$71.61 (37.45)	$61.72 (41.82, 79.60)	$78.65 (67.92)	2.21	0.33
Food preparation costs	$358.50 (239.00, 812.60)	$492.05 (313.46)	$402.27 (119.50, 657.25)	$432.69 (357.66)	$341.44 (100.00, 567.62)	$388.20 (360.15)	0.29	0.86
Lost time from work/school	**$61.77** **(** **0.00, 95.60)**	**$103.08** **(** **170.15)**	**$0.00** **(** **0.00, 59.28)**	**$42.70** **(** **83.85)**	**$0.00** **(** **0.00, 0.00)**	**$0.00** **(** **0.00)**	**6** **.** **70**	**0** **.** **04**

^a^ All costs are presented in Canadian dollars. Bold text denotes statistical significance at *α* = 0.05.

IQR, interquartile range; SD, standard deviation.

#### 3.2.2 Indirect food costs

**Figure 2:**
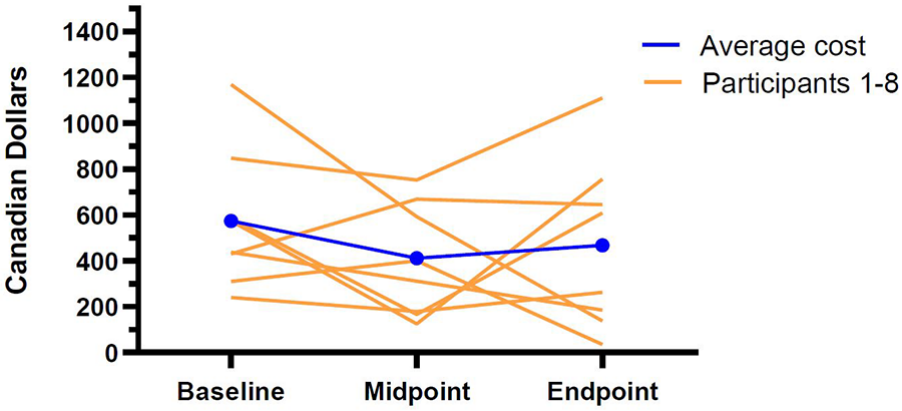
Individual and average indirect food costs at baseline, midpoint, and endpoint.

Like medication costs, total indirect food costs also dropped following the introduction of the intervention, but these differences failed to reach statistical significance (See Figure 2 for an illustration of the indirect food costs at baseline, midpoint, and endpoint for each participant). Much of the apparent decrease appeared to stem from a reduction in food preparation costs (baseline: *M *= $492.05, SD = 313.46; endpoint: *M* = $388.20, SD = $360.15), although a modest decrease in shopping costs was noted as well (baseline: *M *= $99.06, SD = 58.15, endpoint: *M *= $78.65, SD = 67.92).

**Figure 3:**
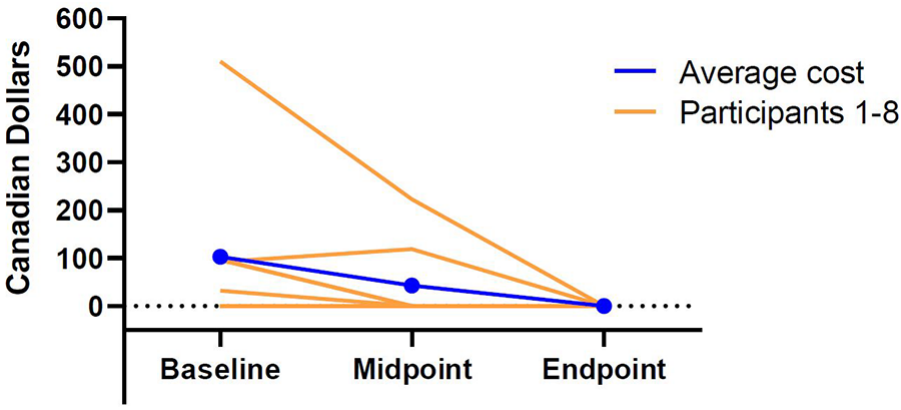
Individual and average costs associated with participants’ lost time from work or school at baseline, midpoint, and endpoint.

In contrast, changes in indirect costs stemming from missed time from work or school did reach statistical significance (Friedman *χ*^2^ = 6.70, *p *= 0.04; See Figure 3 for an illustration of the costs stemming from lost time from work or school at baseline, midpoint, and endpoint for each participant). At baseline, caregivers reported incurring a monthly average of $103.08 (SD = 170.15) in indirect costs as a result of missed time from work or school due to their child's food allergy. At midpoint, however, these costs dropped to an average of $42.70 (SD = 83.85). During the final three months of the study, no participants reported any lost time from work or school. A *post-hoc* Conover-Iman test, corrected for multiple comparisons via Bonferroni's method, revealed caregivers reported incurring significantly fewer costs as a result of lost time from work or school at the endpoint of the study compared to the baseline (*p *= 0.047); however, the remaining comparisons were not statistically significant (baseline vs. midpoint: *p *= 0.36; midpoint vs. endpoint: *p = *0.87).

#### 3.2.3 Food insecurity

**Figure 4:**
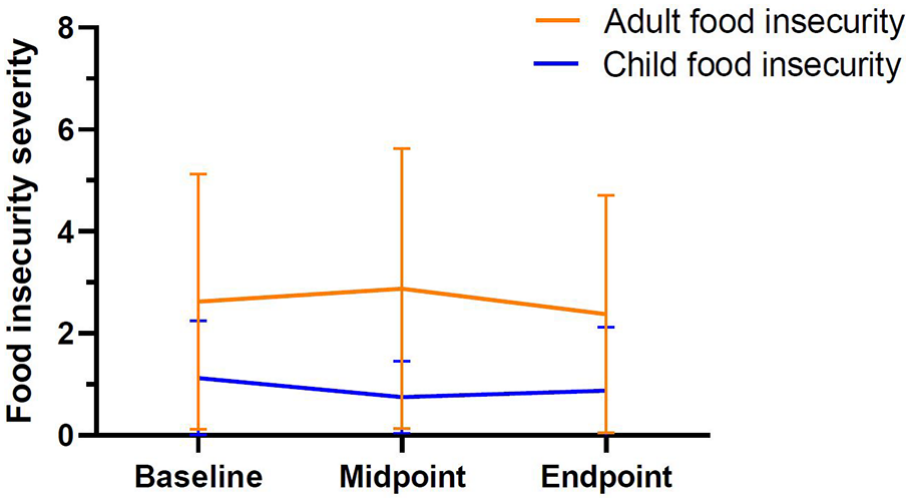
Mean levels of adult and child food insecurity at baseline, midpoint, and endpoint. Scores of two or greater are indicative of food insecurity. Minimum possible child score = 0; minimum possible adult score = 0; maximum possible child score = 8; maximum possible adult score = 10.

At baseline, three of the eight participating families met criteria for moderate child food insecurity (37.5%). By midpoint, child food insecurity appeared to decrease as only one family was found to meet criteria (12.5%). At endpoint, however, the children of three of the eight participating families were once again classified as moderately food insecure (37.5%). By comparison, adult food insecurity appeared slightly more severe in the current sample. At baseline, four families met criteria for moderate adult food insecurity (50%) and one was considered severely food insecure (12.5%). By midpoint, three families were reporting moderate adult food insecurity (37.5%) and two were severely food insecure (25%). At endpoint, three families were still reporting moderate adult food insecurity (37.5%), but only one was classified as severely food insecure (12.5%). While food insecurity appeared to fluctuate to some degree throughout the study, results revealed that the changes in both child (Friedman *χ*^2^ = 2.67, *p *= 0.47) and adult (Friedman *χ*^2^ = 1.50, *p *= 0.26) food insecurity failed to reach statistical significance. Please see Figure 4 for graphical depiction of the changes in adult and child food security throughout the study period among each of the participants.

#### 3.2.4 Psychosocial health

Similar to food insecurity, no statistically significant changes in FA-QoL, nor caregiver mental health (i.e., perceived stress, generalized anxiety, and depression) were noted across the three time-points. Please see Table 3 for a full summary of the findings.

**Table 3:** Pediatric FA-QoL and caregiver mental health scores across the study's duration.

	Baseline		Midpoint		Endpoint		Difference	
	Median (IQR)	Mean (SD)	Median (IQR)	Mean (SD)	Median (IQR)	Mean (SD)	Friedman *χ*^2^	*p*
Pediatric FA-QoL								
Total pediatric FA-QoL	2.91 (1.80, 4.54)	3.06 (1.92)	2.60 (1.20, 3.44)	2.39 (1.33)	3.00 (1.60, 3.80)	2.80 (1.47)	1.78	0.41
Social & dietary restrictions	3.16 (1.80, 4.80)	3.19 (1.93)	3.00 (1.50, 4.60)	3.08 (1.88)	3.44 (1.60, 3.80)	2.92 (1.51)	2.50	0.28
Emotional impact	2.27 (0.08, 3.08)	1.84 (1.56)	1.75 (0.42, 2.50)	1.56 (1.20)	2.60 (0.50, 3.40)	2.31 (2.04)	2.74	0.25
Food anxiety	2.62 (0.33, 3.38)	2.29 (1.97)	2.50 (0.50, 4.50)	2.54 (2.12)	2.33 (0.00, 3.28)	1.90 (1.70)	1.00	0.61
Caregiver mental health								
Perceived stress	19.00 (16.5, 23.5)	19.75 (5.09)	23.00 (17.50, 25.50)	20.12 (8.66)	20.00 (16.50, 23.50)	21.14 (4.88)	0.08	0.96
Generalized anxiety	5.50 (2.50, 8.00)	5.62 (3.38)	8.00 (3.00, 9.50)	6.62 (4.21)	7.00 (4.00, 10.00)	7.00 (4.00)	0.33	0.85
Depression	6.00 (4.00, 24.00)	13.43 (14.54)	18.00 (4.00, 21.00)	13.14 (8.53)	21.50 (11.00, 27.00)	20.17 (13.70)	1.00	0.61

FA-QoL, food allergy quality of life; IQR, interquartile range; SD, standard deviation.

## 4 Discussion

Results from the current study revealed modest, but not statistically significant, increases in direct grocery costs throughout the study period (+3.6% from baseline to endpoint). By comparison, participants reported proportionally larger, but not statistically significant, increases in spending on meals prepared away from home (+120.4% from baseline to endpoint). In contrast to the apparent increase in direct food costs, participants reported non-statistically significant decreases in indirect food shopping (−20.6%) and preparation (−21.1%) costs from baseline to endpoint. Interestingly, participating families were found to incur significantly lower costs related to missed time from school or work due to their child's food allergy at the end of the study compared to baseline (−100%). Despite this welcome finding, results did not provide evidence of significant changes in caregiver mental health or the child's FA-QoL throughout the study period. Similarly, there were no significant changes in food security.

Although the current study did not provide evidence of statistically significant decreases in food costs following the introduction of the food supplement program, it does not mean it was not successful. Over the course of study, Manitoba and other Canadian provinces were experiencing historic levels of inflation, driven, in part, by increased food prices (23, 24). In fact, from February to August 2022 the cost of groceries in Manitoba increased by an estimated 6.9% (25). Despite this, participants in the current study reported only a 3.6% increase in grocery spending over the study period. In light of this finding, it may be argued that the provision of allergen-friendly food played a role in keeping grocery costs below the pace of inflation for participating families. Consistent with this reasoning, a *qualitative* exploration of the same food supplement program assessed in the current study, provided evidence that participants perceived themselves as spending less on groceries in comparison to what they would in the absence of an intervention (22).

It is also worth noting that, for families to be eligible, the child with milk allergy needed to be age <6 years. Given that younger children eat less relative to older children, it is reasonable to speculate that the potential cost savings for a similar intervention, but targeted toward older children, would lead to significant changes. Similarly, it could be argued that the program would have had a larger impact on food costs if the size of the food supplement was adjusted for the number of household members with food allergy. While this was not feasible in the current study, future research should investigate whether adjusting the size of the food supplement to the families’ need provides benefits above and beyond programs with uniform supplements.

In contrast to the moderate increase in grocery costs, participants reported a proportionally larger (+120.4%), but not statistically significant, increase in spending on restaurant and takeaway meals over the study period. While it is difficult to determine what accounted for this change, it is possible that it stems from the fact that the current study was launched during a time when many families were beginning to return to their pre-pandemic activities and routines. During the pandemic, limits on social interaction, in-person work, and non-essential activities, meant many individuals not only lacked opportunities for sharing meals, but also had more time to prepare meals at home (26–29). However, it is likely that as participants returned to some of their pre-pandemic activities, they not only faced greater time pressures, but were also afforded more opportunities for eating out. Given these changes, families may have been more inclined to purchase restaurant and takeaway meals in the later portion of the study following the loosening of public health restrictions (30). Consistent with this reasoning, participants reported sizeable, but non-statistically significant decreases in food preparation costs from baseline to endpoint. Alternatively, as virtually all children had additional food allergies other than milk, it is possible that participants reported increased restaurant spending for themselves but continued to prepare food at home for their child. Such a situation would have resulted in increased restaurant costs, but rather stable food costs, including those associated with allergy-friendly foods beyond milk-free foods.

Interestingly, participants did report significantly lower costs related to missed time from work or school as a result of their child's dairy allergy at the endpoint of the study compared to baseline. Given that the final set of questionnaires were completed near the end of August 2022, it could be argued that the decrease in missed time from work and school reflects a decrease in health care provider visits over the summer months. Over the summer, many healthcare professionals and families take time for vacation which may limit the frequency of visits and consequently lost time from work and school due to food allergy. Alternatively, it may be possible that the decline in absenteeism stems from a decrease in accidental exposures and treatment seeking following the introduction of the supplement program. Previous research indicates that higher income families often prepare an allergen-friendly meal for their entire family; whereas, lower income families are more likely to make a separate allergen-friendly meal for their child with food allergy in order to limit their use of costly allergen-friendly products (22, 31). While this practice likely helps lower grocery costs, it may also increase the likelihood of accidental exposures and increased treatment seeking for acute reactions and exacerbations of allergic comorbidities. Families in the current study, however, may have been encouraged to prepare more allergen-friendly meals for the entire family given that they were provided with allergen-friendly food products free of charge. If this was indeed the case, such a change would arguably decrease the likelihood of exposures and treatment seeking, while also explaining why absenteeism decreased from baseline to endpoint.

Findings from the current study also underscore the need for additional financial support for families with food allergy. Despite the introduction of the food supplement program, household food security (i.e., either child or adult food insecurity) was reported by 50%–62.5% of participating households across the three data collection periods. By comparison, results from the 2019 Canadian Income Survey found 22.4% of families in the lowest income quintile met criteria for food insecurity (32). Naturally, drawing comparisons with the larger Canadian population is made difficult by the small and non-random nature of the current sample, but it is interesting to note that the levels of food insecurity reported herein exceed rates reported in the general population both before and during the pandemic, consistent with American research (8–10).

It is also interesting to note that adult food insecurity appeared to be slightly more prevalent than child food insecurity in the current study. Findings from the qualitative arm of the current project (presented in a separate publication) suggest that this discrepancy is rooted in the fact that mothers tend prioritize the dietary needs of their children when faced with scarcity (22). While similar findings have been noted in the context of food insecurity more generally, they have only been described among families managing food allergy recently.

In light of these findings, it is apparent that gaps remain in the resources available to families struggling with the high costs of allergen-friendly diets. Arguably, future researchers, policy makers, and non-profits all have a role to play in filling this gap; however, food manufacturers can also make meaningful strides in reducing food insecurity through the greater adoption of allergen-safe manufacturing practices and responsible use of precautionary allergen labels (i.e., PALS). To date, PALS have been criticized by consumers as lacking credibility and contributing to confusion regarding their true level of risk (33, 34). Rather than using precautionary allergen labels as a means of reducing their liability, food manufacturers are encouraged to combine allergen-safe manufacturing practices with the judicious use of PALS in order to provide consumers with a wider range of safe and affordable products.

Several limitations of the current study should be noted. Despite our best efforts, we were only able to recruit 11 participants, three of which, were lost to follow-up. Because of the small sample size, we were unable to assess the normality of our data, which necessitated the use of non-parametric tests. For these reasons, the current study was likely only powered to detected large changes in the outcome variables across the three data collection points. While the recruitment difficulties of the current study were disappointing, they were not wholly surprising as research suggests many individuals are reluctant to accept food charity due to stigma and the shame involved (35). In light of this finding, future interventions aimed at addressing food insecurity among families managing food allergy should think carefully about how to minimize the impact of stigma on the intervention's uptake.

Beyond its sample size, the study was also arguably limited by its lack of a control condition or control group. In the absence of these design features, we were unfortunately unable to control for the effects of several important social and economic changes that coincided with the launch of the study, including a loosening of a number of pandemic-related public health restrictions in the province where the study took place (30). Over the same period, Canada and many other countries were experiencing historic levels of inflation, driven in part by rising food prices (23, 24). Because these changes coincided with the launch of the study, it is likely that they played some role in in obscuring the impact of the food supplement program on food costs. In light of this limitation, future evaluations of similar programs should make use of randomized control trials or randomized crossover designs to ensure the casual link between the program and the outcomes can clearly be made.

Lastly, because the study was limited to households with a young child with a dairy allergy, it is not clear whether the results generalize to other food allergens and ages. In light of these limitations, there remains a need to determine whether and under what conditions, food supplement programs, like the one evaluated in the current study, help to reduce the financial burden of food allergy.

## 5 Conclusion

Findings from the current study suggest that a novel food supplement program aimed at families with a young child with dairy allergy helped to keep grocery costs below the pace of inflation. Moreover, the introduction of the program also coincided with a reduction in the number of days caregivers lost from work or school as a result of their child's food allergy. Despite these encouraging results, many participants continued to report relatively high levels of food insecurity. In light of this finding, it appears as though more financial supports are needed for lower income families managing food allergy.

## Data Availability

The raw data supporting the conclusions of this article will be made available by the authors, without undue reservation.
